# Witnessing extinction in real time

**DOI:** 10.1371/journal.pbio.2003080

**Published:** 2018-02-06

**Authors:** Karen R. Lips

**Affiliations:** University of Maryland, Department of Biology, College Park, Maryland, United States of America

This Perspective is part of the C*onservation Stories from the Front Lines Collection*

I started my amphibian research a little like Thoreau, living alone in a cabin in the woods and recording the seasonal variation in the natural world. I lived in a shack without plumbing or electricity, an hour away from the closest house, in a cloud forest on the top of a mountain that straddled the border between Costa Rica and Panama. I scrambled along mountain streams chasing seasonal reproductive data on treefrogs. The species I studied, *Isthmohyla calypsa*, remains one of the most spectacular frogs I have ever seen; adults were a brilliant, iridescent green with a bright white throat pouch, and their skin was as textured as the spikey moss they lived on, apparently camouflaging them from predators [1].

I was living a field biologist’s dream and expected to spend my career studying tropical montane amphibians in one of the most beautiful and least studied regions in the world. I couldn’t know that my study site in this remote cloud forest would give me a front row seat to one of the most distressing ecological mysteries of our time.

As a graduate student, I had heard the ominous reports describing the mysterious disappearances of amphibian populations around the world. At the time, scientists were debating whether this was a real phenomenon, what might cause it, and whether these events were connected. The large number of species affected was especially worrisome; of the suspected threats, none typically caused extirpation or extinction of multiple species while leaving the habitat intact. It was more than a search for a needle in a haystack—we were still debating the existence of the haystack. I was just a graduate student, and while I wasn’t actively looking for the needle, I would step right on it.

That was triggered with my intent to capture the early breeding season of an interesting toad (*Incilius fastidiosus*). These toads would emerge from underground to lay rosary-like strands of eggs amidst torrential downpours that turned the pastures into swampy meadows. I returned to my cloud forest site weeks earlier than usual, at the beginning of the rainy season in 1993, to continue some preliminary work from the previous year. But the toads never came out.

I knew that a related species—the golden toad (*Incilius periglenes*)—had recently disappeared from its entire range just a few hundred miles north of me. But I was soon focused on other, more immediate concerns as I realized that it wasn’t just the toads that were gone, my mossy green treefrogs and the other stream frogs were also missing. I blamed the weather, my technique, the bright light of my headlamp—I was grasping at straws and I knew it, but I couldn’t really believe that this might be another “enigmatic decline.” Maybe it would have been different if I had found hundreds of dead frogs, but they had disappeared when I wasn’t looking. When I returned in 1996, the streams were dead silent—no frogs were calling, species were missing, overall abundance was down by 90%, and even the gelatinous egg masses were gone. I spent that entire summer arguing with myself about what would cause the loss of so many different kinds of amphibians, so quickly, and without any obvious change to the habitat. By the time the semester began in September, I was back in my office ready to present the argument that this site in southern Costa Rica was the most recent instance of an enigmatic amphibian decline site [2].

Writing up that manuscript forced me to seriously consider the possibility that what was happening in Costa Rica was part of a larger global phenomenon. As I compared what we knew of the *I*. *periglenes* disappearance in the north to what I had documented with *I*. *fastidiosus* and all the stream frogs in the south, I hypothesized that we were watching a wave sweeping through these ecosystems, and I wanted to get out ahead of it. I set up a new camp farther south, this time in western Panama, to see what might come next. Honestly, I did not really expect that this would happen or that I would be able to document it if it did. I expected to be able to study these new populations for a long time. The sad truth is I had nothing to lose. There were not enough frogs to do any meaningful ecological study at my Costa Rican site.

This next site, Fortuna, was another protected area in the cloud forest. It was a spectacular landscape at the top of the watershed in a high river valley. There was a field station with electricity and hot showers, accessed by a paved highway that crossed dozens of streams. I had a list of species known from the site and descriptions of habitats from historical biological surveys. I was thrilled to have a new site with many frogs, and I immediately set up new transects to begin quantifying seasonal variation in community richness, reproductive diversity, and population abundance for the 40-some species at this site.

The joy would be short-lived. In December 1996, I returned to this site and realized that things were not normal. Frog numbers were not quite what they were before, with some streams apparently fine but others with much lower abundance. The weather was perfect for frogs, and the habitat was intact in this protected area. I saw no evidence that anything was obviously different. But many of the animals we were catching were not healthy, often expiring in our hands after a short struggle to escape. Over the next few weeks, we found dozens of dead or dying animals on our transects. We still had no idea what was killing the frogs, but we finally had bodies to examine that we hoped would show us if they were dying of an infectious disease, pollution, or something else. Could this be the smoking gun? Was this what happened to the golden toad? To my sparkling green treefrogs in Costa Rica?

Eventually, we would find out that these animals were infected by a microscopic chytrid fungus (*Batrachochytrium dendrobatidis*) that would soon be described by Dr. Joyce Longcore and colleagues from studying animals that died at the United States National Zoo and that it was the same fungus found in dying Australian frogs [3–5]. This was the evidence we needed to show that a fungal pathogen was involved in many declining amphibian populations all around the globe. The haystack was real! We had found the needle!

In Central America, I now had three locations that all showed the same pattern of decline, and with some additional study of the few dead frogs I found in Costa Rica, we were able to show that this fungus had also been present in two of those populations just before they disappeared [6]. We did not yet have evidence of the fungus in the golden toad or frogs from other sites in Costa Rica but we were able to show across several sites that patterns of decline among communities were similar and that closely related species also showed similar patterns of decline. All of this suggested that the cause of the declines was the same and that this was a regional problem [7].

I was finally convinced that this skin fungus was responsible for the losses of amphibians across Costa Rica and western Panama, and I was pretty sure it was moving as an epidemic wave. But we did not have eyewitness accounts of the older declines to the north and no forensic evidence that the fungus had arrived at those sites prior to population declines. We would have to try to catch it again, and we set up transects at a new site at El Cope in central Panama. This was a cloud forest site in another national park a few hundred kilometers to the east of Fortuna with even more species of amphibians. Every spring as we prepared to return to central Panama and run the transects for another field season, we wondered, would the frogs be there? Or would this be the year that the fungus arrived? Would we be able to detect the earliest symptoms? Would this site be different somehow? Were the epidemics over?

We spent six years waiting for something to happen. In September 2004, two months after I returned from our latest stakeout at El Cope, my graduate students called me with the news that they were finding dead frogs on the transects. It was happening! It was simultaneously exhilarating and horrifying to realize that we were right: the fungus was moving! It was an epidemic! And yet…..we couldn’t stop it.

I immediately felt panic—and we talked about how to change the sampling plans to get as much data as possible. We hired extra people to survey all the transects every day to collect as many dead frogs as possible so we could compare the changes in the amphibians in the streams to those in the forest. By January, we were not finding many frogs at all; we had gone from needing five hours to survey all the animals in one 200-meter transect to needing only one hour to survey all the animals in two or three transects [8–9].

It was also frustrating to be trapped at school while “my” frogs were dying in Panama—after all those years of work and all those transects, I would miss the actual epidemic. But I knew that while my field team documented the devastation, my responsibility was to tell everybody in the larger community what was happening and what it meant. In November, I described the die-off happening in Panama to our larger amphibian disease research group to get their input on what this meant and what we could or should do. I talked with my colleagues in the conservation community about this, and we discussed what could be done now that we knew that this fungus would continue to move through Panama and elsewhere [10]. This event would set off years of meetings and presentations and discussions with policymakers, conservation leaders, and scientists as to how we should plan for long-term research and conservation actions.

As the years passed, the information spread, scientists made progress on many fronts, and I had more time to think about what we had discovered. I realized that this was not just population declines but that what we had witnessed was the extinction of many species. It might take 50 years to confirm it [11], but the massive epidemic coupled with the small ranges and low abundances of these tropical species was sure to cause extinction of many of these rainforest jewels.

I found that amazing and jarring. Extinction is supposed to be a rare event—and yet we had documented multiple cases from the least likely places—remote, protected areas with extremely high biodiversity. Noticing losses and mourning changes in our natural world is a sadness shared with all peoples; what is different for biologists is that we are distinctively positioned to catalogue the decline of species and to understand the true scope of it.

First-hand experiences always make a greater impact, and I have found it hard to communicate just how bad the situation at my sites is to those who haven’t been there. Having extensive data that showed changes in population numbers was critical for convincing the scientific community of the extent of the problem and that extinction was really occurring. The dramatic image showing the precipitous decline in the amphibians of El Cope based on the hundreds of transects we surveyed convinces everybody of the seriousness of this disease. They are impressed when we show them the spread of the fungus across the map and the huge extent of the problem across the most diverse communities of the Americas [12]. We can even describe the impacts on all the other components of these ecosystems—the loss of the predatory snakes, the large patches of filamentous green algae where the tadpoles have been lost, changes in the stream-dwelling insects, and even the water chemistry [13].

But it is impossible to convey what this means to people who have not seen it for themselves. I will always remember the reaction of our Costa Rican colleagues who came to visit us in El Cope. They were relatively young researchers, and so while they were fully aware of the problem and had studied amphibians for years in their country, they were too young to have seen the golden toad or the harlequin frogs and only knew the post-decline world. So, when they saw the many gleaming, sparkling, colorful amphibians scattered about the streams, trails, and vegetation of El Cope, they responded like kids at Christmas, stumbling about in disbelief, oblivious to everything else, marveling at the diversity and abundance of amphibians. It broke my heart to tell them that this was what Costa Rica used to be like, but I was glad they got to see what was normal was before it too disappeared.

I look at the folders of data in my office and think about the descriptions and field guides we started that don’t seem very useful anymore. I often joke that I no longer work in Central America because I can’t find any frogs—but it’s not really a joke. Most places I know have a fraction of the amphibians that they used to—and going back is a lesson in frustration. Counting those zeros is helpful in establishing the lack of recovery, but who wants to spend their lives documenting that the frogs are still gone? It’s exhausting, and eventually one has to disengage and if not move on, then at least refocus on something more productive.

These days, I am thinking of how to make a difference on a larger scale by working on environmental policy issues and getting involved with international education and science diplomacy. Recently, I have been working with colleagues in the science and policy arena to find a way to reduce the threat of importing new wildlife diseases for live amphibians [14]. The many years of hard work by the scientific community on *B*. *dendrobatidis* provided a solid foundation for US Fish and Wildlife Service to justify limiting imports of some species of salamanders that might introduce a new species of salamander-specific chytrid fungus (*B*. *salamandrivorans*) to the US [15]. In collaboration with the Leopold Leadership Program, COMPASS, and the National Socio-Environmental Synthesis Center (SESYNC), we have developed my experience into a case study on environmental policy and leadership that is available online [16] for educators to use.

Last year I served as a Jefferson Science Fellow at the US Department of State (Bureau of Western Hemisphere Affairs in the Office of Public Diplomacy and Public Affairs). It might seem curious that a biologist is at the State Department, but science diplomacy, international collaborations, and scientific exchanges are essential for addressing many science and technology issues, including amphibian population declines. As a Jefferson Fellow, I met with environmental decision-makers, participated in educational exchange programs, and shared my research with students, researchers, citizen groups, and decision-makers from across Latin America and the Caribbean. It was especially rewarding to connect with students and educators from Latin America who work at schools or museums to talk about science, technology, engineering, and mathematics (STEM) careers and the importance of science and science communication because it complemented my American Association for the Advancement of Science (AAAS) Leshner Fellowship goals of increasing public engagement with science.

Change happens quickly these days, and many things that seemed impossible have come to pass. I’m hoping we see a return to the high-diversity and high-abundance communities of tropical amphibians in my lifetime. Wouldn’t that be great?

## Abbreviations

AAAS: American Association for the Advancement of Science
SESYNC: National Socio-Environmental Synthesis Center
STEM: science, technology, engineering, and mathematics

## References

[1] Lips KR. Reproductive trade-offs and bet-hedging in *Hyla calypsa*, a Neotropical treefrog. 2001; Oecologia 128: 509–518. doi: 10.1007/s004420100687 28547396 10.1007/s004420100687

[2] Lips KR. Decline of a Tropical Amphibian Fauna. 1998; Conserv. Biol. 12(1): 106–117.

[3] Lips KR. Mass mortality of the anuran fauna at an upland site in Panama. 1999; Conserv. Biol. 13(1): 117–125.

[4] Berger L, Speare R, Daszak P, Green DE, Cunningham AA, Slocombe R, et al Chytridiomycosis causes amphibian mortality associated with population declines in the rainforests of Australia and Central America. Proc. Natl. Acad. Science. 1998; 95: 9031–9036. 10.1073/pnas.95.15.9031 PMC21197 9671799

[5] Lips K. What if there is no happy ending? Science communication as a path to change. 5 May 2013. In: Scientific American Guest Blog. https://blogs.scientificamerican.com/guest-blog/what-if-there-is-no-happy-ending-science-communication-as-a-path-to-change/. Accessed on 22 June 2017.

[6] Lips KR, Green DE, Papendick R. Chytridiomycosis in wild frogs from southern Costa Rica. 2003; J. Herpetol. 37(1): 215–218.

[7] Lips KR, Reeve JD, Witters L. Ecological traits predicting amphibian population declines in Central America. 2003; Conserv. Biol. 17(4): 1078–1088.

[8] Lips KR, Brem F, Brenes R, Reeve JD, Alford RA, Voyles J, et al Infectious disease and global biodiversity loss: pathogens and enigmatic amphibian extinctions. 2006; Proc. Natl. Acad. Science. 103(9): 3165–3170. 10.1073/pnas.0506889103 PMC1413869 16481617

[9] Crawford AJ, Lips KR, Bermingham E. Epidemic disease reduces evolutionary diversity of amphibians. 2010 Proc. Natl. Acad. Science. 107(31): 13777–13782. 10.1073/pnas.0914115107 PMC2922291 20643927

[10] Mendelson JR III, Lips KR, Gagliardo RW, Rabb GB, Collins JP, Diffendorfer JE, et al Confronting amphibian declines and extinctions. 2006; Science 313: 48 doi: 10.1126/science.1128396 16825553 10.1126/science.1128396

[11] IUCN 2017. The IUCN Red List of Threatened Species. Version 2017–1. <http://www.iucnredlist.org>. Downloaded on 12 May 2017. http://www.iucnredlist.org/static/categories_criteria_3_1#introduction. Accessed on 22 June 2017.

[12] Lips KR, Diffendorfer J, Mendelson J, Sears M. Riding the Wave: Reconciling the roles of disease and climate change in amphibian declines. 2008; PLoS Biol 6(3): e72 doi: 10.1371/journal.pbio.0060072 18366257 10.1371/journal.pbio.0060072 PMC2270328

[13] Whiles M, Hall RO Jr, Dodds WK, Verburg P, Huryn AD, Pringle CM, et al Disease-driven amphibian declines alter ecosystem processes in tropical streams. 2012; Ecosystems.

[14] Martel A, Blooi M, Adriaensen C, Van Rooij P, Beukema W, Fisher MC, et al Recent introduction of a chytrid fungus endangers western Palearctic salamanders. 2014; Science 10.1126/science.1258268 10.1126/science.1258268 PMC5769814 25359973

[15] US Fish and Wildlife Service. Department of the Interior. 2016. 50 CFR Part 16, RIN 1018–BA77. Docket No. FWS–HQ–FAC–2015–0005; FXFR13360900000–156–FF09F14000. Injurious Wildlife Species; Listing salamanders due to risk of salamander chytrid fungus. Federal Register 81 (8).

[16] Krebs M, Lips K, McIntosh T, Nevle R, Sturner P. Stopping wildlife disease from becoming a crisis: a collaborative leadership success story. 2015; SESYNC Case Study for Teaching Socio-Environmental Synthesis. https://www.sesync.org/stopping-a-wildlife-disease-from-becoming-a-crisis-a-collaborative-leadership-success-story-2016-5. Accessed on 22 June 2017.

